# Supplementary material for: Antigenic cartography using hamster sera identifies SARS-CoV-2 JN.1 evasion seen in human XBB.1.5 booster sera
Source: bioRxiv. 2024 Apr 6:2024.04.05.588359. Preprint. [Version 1] doi: 10.1101/2024.04.05.588359 (PMC11071293; doi:10.1101/2024.04.05.588359)
Supplement: Supplement 2 — Table S1. Antigenic distances from variants to D614G. 95% confidence intervals were estimated by building maps with only a subset of the data and measuring the distribution of distances between each D614G and each virus across these maps. Table S2. Demographic information of multiple antigen exposure sera. Table S3. Residual titers for all antigens in each multiple antigen exposure serum group. Table S4. Viruses used for hamster infections and spikes used for pseudoviruses. Table S5. Demographic information of primary infection serum samples from the EPICC study. Table S6. Demographic information of primary infection serum samples commercially obtained. Table S7. SARS-CoV-2 variant spikes of human primary infection serum samples from the EPICC study. Table S8. SARS-CoV-2 variant spikes of human primary infection serum samples commercially obtained. [file media-2.pdf]

Table S1. Antigenic distances from variants to D614G

	Human map (n=84)	Hamster map (n=82)	Merged human-hamster map (n=166)
**Alpha**	0.6 (0.4 – 0.7)	0.9 (0.6 – 1.3)	0.7 (0.6 – 0.9)
**Beta**	3.8 (3.7 – 3.9)	3.6 (3.3 – 3.8)	3.6 (3.4 – 3.7)
**Gamma**	3.3 (3.2 – 3.4)	2.1 (1.8 – 2.4)	3.1 (3.0 – 3.2)
**Iota**	2.3 (2.2 – 2.5)	2.1 (1.8 – 2.3)	2.1 (2.0 – 2.3)
**Epsilon**	0.6 (0.5 – 0.8)	0.2 (0.0 – 0.5)	0.3 (0.2 – 0.4)
**Delta**	1.5 (1.4 -1.6)	1.2 (1.0 – 1.4)	1.4 (1.3 – 1.5)
**Lambda**	1.3 (1.2 – 1.5)	-	1.1 (0.9 – 1.2)
**Mu**	3.6 (3.5 – 3.8)	-	3.4 (3.3 – 3.6)
**BA.1**	**7.0 (6.9 – 7.2)**	**5.7 (5.5 – 6.0)**	6.3 (6.2 – 6.5)
**BA.1.1**	7.3 (7.2 – 7.5)	-	7.0 (6.8 – 7.1)
**BA.2**	6.4 (6.2 – 6.6)	5.6 (5.3 – 5.9)	6.0 (5.8 – 6.2)
**BA.2.12.1**	6.4 (6.3 -6.6)	-	6.1 (6.0 – 6.3)
**BA.4/BA.5**	**5.9 (5.8 – 6.1)**	**6.6 (6.3 – 7.0)**	6.3 (6.1 – 6.5)
**XBB**	-	7.2 (6.9 – 7.6)	7.4 (7.1 – 7.7)
**XBB.1.16**	-	7.7 (7.4 – 8.0)	7.9 (7.7 – 8.3)
**XBB.1.5**	-	6.7 (6.5 – 7.0)	7.0 (6.9 – 7.2)
**XBB.2.3**	-	7.4 (7.1 – 7.8)	7.6 (7.3 – 8.0)
**EG.5**	-	7.3 (7.0 – 7.5)	7.5 (7.3 – 7.8)
**EG.5.1**	-	7.1 (7.2 – 8.4)	7.3 (7.1 – 7.6)
**JN.1**	-	6.4 (6.1 – 6.7)	6.6 (6.3 – 6.9)

Antigenic distances (antigenic units) between D614G and variants from human primary infection map, hamster primary infection map and merged human-hamster map. An antigenic distance equivalent to one is represented by one grid-square on the antigenic map. 95% confidence intervals for antigenic distances were calculated.

Table S2. Demographic information of multiple antigen exposure sera from the PASS study

	Overall (n=123)[a]	V3 (n=39)	V3 + Bi (n=19)	V3 + PVI (n=25)	V4 (n=16)	V4 + Bi (n=8)	XBB.1.5 booster (n=16)[c]
**Age group**							
18-44	57 (46.3%)	19 (48.7%)	15 (78.9%)	12 (48.0%)	2 (12.5%)	0 (0%)	9 (56.3%)
45-64	63 (51.2%)	19 (48.7%)	4 (21.1%)	13 (52.0%)	13 (81.3%)	7 (87.5%)	7 (43.7%)
65+	3 (2.5%)	1 (2.6%)	0 (0%)	0 (0%)	1 (6.2%)	1 (12.5%)	0 (0%)
Median age (IQR)	46.0 (38.0 - 54.0)	45.0 (35.0 - 52.0)	39.0 (34.5 - 43.0)	45.0 (38.0 - 53.0)	55.5 (51.0 - 58.0)	54.5 (53.5 - 59.3)	41.5 (37.8 - 50.5)
**Gender**							
Female	82 (66.7%)	25 (64.1%)	11 (57.9%)	18 (72.0%)	10 (62.5%)	6 (75.0%)	12 (75.0%)
Male	41 (33.3%)	14 (35.9%)	8 (42.1%)	7 (28.0%)	6 (37.5%)	2 (25.0%)	4 (25.0%)
**Race/Ethnicity**							
White	80 (65.0%)	25 (64.1%)	14 (73.7%)	17 (68.0%)	9 (56.3%)	6 (75.0%)	9 (56.3%)
Black	15 (12.2%)	4 (10.3%)	2 (10.5%)	3 (12.0%)	2 (12.5%)	1 (12.5%)	3 (18.7%)
Hispanic or Latino	7 (5.7%)	1 (2.6%)	0 (0%)	2 (8.0%)	3 (18.8%)	1 (12.5%)	0 (0%)
Others	21 (17.1%)	9 (23.1%)	3 (15.8%)	3 (12.0%)	2 (12.5%)	0 (0%)	4 (25.0%)
**Charlson Co-morbidity Index**							
0	106 (86.2%)	34 (87.9%)	17 (89.5%)	21 (84.0%)	13 (81.2%)	5 (62.5%)	16 (100%)
1	9 (7.3%)	2 (5.1%)	2 (10.5%)	3 (12.0%)	1 (6.2%)	1 (12.5%)	0 (0%)
2	5 (4.0%)	2 (5.1%)	0 (0%)	1 (4.0%)	1 (6.2%)	1 (12.5%)	0 (0%)
3	3 (2.5%)	1 (2.6%)	0 (0%)	0 (0%)	1 (6.2%)	1 (12.5%)	0 (0%)
**Primary vaccine (V1+V2)**							
Pfizer BNT162b2	123 (100%)	39 (100%)	19 (100%)	25 (100%)	16 (100%)	8 (100%)	16 (100%)
**First booster (V3)**							
Pfizer BNT162b2	120 (97.5%)	39 (100%)	19 (100%)	24 (96.0%)	15 (93.8%)	8 (100%)	15 (93.7%)
Moderna mRNA-1273	3 (2.5%)	0 (0%)	0 (0%)	1 (4.0%)	1 (6.2%)	0 (0%)	1 (6.3%)
**Second booster (V4)**							
Pfizer BNT162b2	22 (17.9%)	-	0 (0%)	-	13 (81.2%)	7 (87.5%)	2 (12.5%)
Pfizer bivalent (original and Omicron BA.4/BA.5)	31 (25.2%)	-	19 (100%)	-	0 (0%)	0 (0%)	12 (75.0%)
Moderna mRNA-1273	4 (3.3%)	-	0 (0%)	-	3 (18.8%)	1 (12.5%)	0 (0%)
Moderna bivalent (original and Omicron BA.4/BA.5)	1 (0.8%)	-	-	-	-	-	1 (6.3%)
Pfizer monovalent XBB.1.5	1 (0.8%)	-	-	-	-	-	1 (6.3%)
Unboosted	64 (52.0%)	-	0 (0%)	-	0 (0%)	0 (0%)	0 (0%)
**Third booster (V5)**							
Pfizer bivalent (original and Omicron BA.4/BA.5)	10 (8.1%)	-	-	-	-	8 (100%)	2 (12.5%)
Pfizer monovalent XBB.1.5	12 (9.8%)	-	-	-	-	-	12 (75.0%)
Moderna monovalent XBB.1.5	1 (0.8%)	-	-	-	-	-	1 (6.3%)
Unboosted	100 (81.3%)	-	-	-	-	-	1 (6.3%)
**Fourth booster (V6)**							
Pfizer monovalent XBB.1.5	2 (1.6%)	-	-	-	-	-	2 (12.5%)
Unboosted	121 (98.4%)	-	-	-	-	-	14 (87.5%)
**Days between most recent vaccination and serum sample collection**							
Median (IQR)	39.0 (27.0 - 61.0)	43.0 (33.5 - 53.5)	29.0 (27.5 - 35.0)	169.0 (148.0 - 267.0)	33.0 (22.5 - 53.0)	37.0 (32.8 - 43.0)	21.5 (16.0 - 27.3)
**Days between infection symptom onset and serum sample collection**							
Median (IQR)	70.5 (38.8 - 184.0)	-	-	56.0 (37.0 - 75.0)	-	-	553.0 (446.5 - 672.0)
**Infecting genotype**[b]							
BA.1.1	3 (2.5%)	-	-	2 (8.0%)	-	-	1 (6.3%)
BA.1.18	1 (0.8%)	-	-	-	-	-	1 (6.3%)
BA.1.19	1 (0.8%)	-	-	1 (4.0%)	-	-	-
AY.25	1 (0.8%)	-	-	1 (4.0%)	-	-	-

[a]Samples collected from PASS study; 29 participants contributed serum samples collected at different timepoints to 2 or 3 groups (3 contributed to V3 and V4, 3 contributed to V3 and V3+Bi, 6 contributed to V3 and V3+PVI, 1 contributed to V3 and V4+Bi, 3 contributed to V3 and XBB.1.5 booster, 4 contributed to V4 and V4+Bi, 3 contributed to V3+Bi and XBB.1.5 booster, 2 contributed to V3+PVI and XBB.1.5 booster, 1 contributed to V3, V4 and V4+Bi, 1 contributed to V3, V4+Bi and XBB.1.5 booster, and 2 contributed to V3, V3+PVI and XBB.1.5 booster)

[b]Genotypes assigned based on Pango 4.1.2

IQR, Interquartile range; V1+V2, 2 doses ancestral mRNA vaccine; V3, 3 doses ancestral mRNA vaccine; V3+Bi, 3 doses ancestral + 1 dose bivalent mRNA vaccine; V4, 4 doses ancestral mRNA vaccine; V4+Bi, 4 doses ancestral + 1 dose bivalent mRNA vaccine

[c]XBB.1.5 booster group includes six individuals without reported PVI, three individuals with presumed BA.1 PVIs and four individuals with presumed BA.5 PVIs after three doses of the ancestral COVID-19 vaccine, and three individuals with presumed XBB PVIs after the bivalent booster.

Table S3. Residual titers for all antigens in each multiple antigen exposure serum group

	**V3 + bivalent** (n = 19)	**V3 + PVI** (n = 23)	**V4** (n = 16)	**V4 + bivalent** (n = 8)	**XBB 1.5 Booster** (n = 16)
**D614G**	0.48	0.26	0.36	0.36	0.23
**Beta**	1.49	1.72	1.41	1.3	0.23
**Delta**	0.71	0.93	0.73	0.67	-0.01
**BA.4/BA.5**	2.45	2.65	2.2	2.26	1.66
**XBB.1.16**	0.32	0.19	0.18	0.41	0.67
**XBB.1.5**	-1.5	-1.6	-1.46	-1.4	0.45
**XBB.2.3**	0.25	0.26	0.26	0.42	0.74
**XBB**	-0.03	-0.15	0.01	-0.21	-0.73
**EG.5.1**	-0.25	-0.4	-0.5	-0.61	1.06
**JN.1**	-1.59	-1.7	-1.46	-1	-1.95

Residual titers are calculated by subtracting the predicted titer (from fitting the landscape) from the measured titer and are represented as log titers.
Positive residual titers indicate that measured titer was greater than predicted titer. Negative residual titers indicate that measured titer was less than predicted titer.

Table S4. Viruses used for hamster infections and spikes used for pseudoviruses.

Virus used for hamster infection				Spike used in pseudovirus	
Name	Strain	Source	GenBank or GISAID #	Name	GenBank or GISAID #
WT	hCoV-19/USA-WA1/2020	BEI, NR-52281	MN985325.1	D614G	EPI_ISL_5851484
B.1.1.7	hCoV-19/USA/CA_CDC_5574/2020	BEI, NR-54011	MW981411	B.1.1.7	MW422256
P.1	hCoV-19/Japan/TY7-503/2021	BEI, NR-54984	OK091603	P.1	MW520923
B.1.427	hCoV-19/USA/CA-CDC-48018/2020	BEI, NR-55338	MZ376661	B.1.427	MZ376661
B.1.526	hCoV-19/USA/NY-NP-DOH1/2021	BEI, NR-55637	EPI_ISL_1080761	B.1.526	MW519672
B.1.617.2	hCoV-19/USA/PHC658/2021	BEI, NR-55612	OR074942	B.1.617.2	MW934201
BA.1	hCoV-19/USA/MD-HP20874/2021	BEI, NR-56461	OQ361639	BA.1	OQ361639
BA.1.1	hCoV-19/USA/GA-EHC2811C/2021	BEI, NR-56482	EPI_ISL_7171744		
BA.2	hCoV-19/USA/MD-HP24556/2022	BEI, NR-56512	ON128736	BA.2	ON128736
BA.5	hCoV-19/ USA/COR-22-063113/2022	BEI, NR-58616	ON972631	BA.4/5	EPI_ISL_12464782
XBB.1.5	hCoV-19/USA/MD-HP40900/2022	BEI, NR-59104	EPI_ISL_16026423	XBB.1.5	EPI_ISL_15687648
XBB.1.16	hCoV-19/USA/CA-Stanford-139_S23/2023	BEI, NR-59442	EPI_ISL_17417328	XBB.1.16	EPI_ISL_17717392
				XBB.2.3	EPI_ISL_16382405
				BQ.1.1	EPI_ISL_16364753
XBB	hCoV-19/USA/CA-Stanford-109_S21/2022	BEI, NR-58925	EPI_ISL_15509864	XBB	EPI_ISL_16160901
B.1.351	hCoV-19/South Africa/KRISP-K005325/2020	BEI, NR-54009	EPI_ISL_678615	B.1.351	MW598419
				B.1.621	MZ232908
				BA.2.12.1	OR366997
				EG.5	OQ873579
EG.5.1	hCoV-19/USA/MD-HP47946/2023	BEI, NR-59503	EPI_ISL_17738077	EG.5.1	EPI_ISL_17738077

Table S5. Demographic information of primary infection serum samples from the EPICC study

	Overall (n=45)[a]
**Age group**	
<18	5 (11.1%)
18-44	20 (44.4%)
45-64	15 (33.3%)
65+	5 (11.1%)
**Gender**	
Female	19 (42.2%)
Male	26 (57.8%)
**Race/ Ethnicity**	
White	21 (46.7%)
Hispanic or Latino	14 (31.1%)
Black	7 (15.6%)
Others	3 (6.7%)
**Severity of initial infection**	
Hospitalized	23 (51.1%)
Outpatient	22 (48.9%)
**Charlson comorbidity index**	
0	19 (42.2%)
1-2	17 (37.8%)
3-4	6 (13.3%)
>5	3 (6.7%)
**Primary vaccine**	
Unvaccinated	45 (100.0%)
**Days between infection symptom onset and sera sample collection**	
Median ± SD (range)	27 ± 9.7 (8.0 - 51.0)
**Infecting genotype[b]**	
B.1	10 (22.2%)
B.1.2	6 (13.3%)
BA.1	3 (6.7%)
B.1.1.207	3 (6.7%)
B.1.1.7	3 (6.7%)
AY.100	2 (4.4%)
AY.14	2 (4.4%)
AY.25	2 (4.4%)
B.1.617.2	2 (4.4%)
B.1.429	2 (4.4%)
AY.119	1 (2.2%)
AY.25.1	1 (2.2%)
AY.3	1 (2.2%)
AY.44	1 (2.2%)
AY.47	1 (2.2%)
AY.62	1 (2.2%)
AY.74	1 (2.2%)
B.1.1.519	1 (2.2%)
B.1.526	1 (2.2%)
P.1.10	1 (2.2%)

[a] *Sample collected from EPICC study*

[b] *Genotypes assigned based on Pango 4.0.6*

Table S6. Demographic information of primary infection serum samples commercially obtained

	Overall (N=31)
**Gender**	
Female	10 (32.3%)
Male	21 (67.7%)
**Infecting genotype**	
B.1	2 (6.5%)
B.1.1.7	7 (22.6%)
B.1.2	2 (6.5%)
B.1.234	1 (3.2%)
B.1.429	3 (9.7%)
B.1.577	1 (3.2%)
C.11	1 (3.2%)
C.37	10 (32.3%)
P.1	4 (12.9%)
**Time between infection symptom onset and sera sample collection**	
Median ± SD (range)	5 ± 12.6 (2.0 - 59.0)

Table S7. SARS-CoV-2 variant spikes of human primary infection serum samples from the EPICC study

SampleID	Pangolin 4.0.6	Spike Substitutions	Spike Deletions	Accession
Conv-15	BA.1	S:A67V,S:T95I,S:Y145D,S:L212I,S:G339D,S:S371L,S:S373P,S:S375F,S:K417N,S:N440K,S:G446S,S:S477N,S:T478K,S:E484A,S:Q493R,S:G496S,S:Q498R,S:N501Y,S:Y505H,S:T547K,S:D614G,S:H655Y,S:N679K,S:P681H	S:H69-,S:V70-,S:G142-,S:V143-,S:Y144-,S:N211-	SAMN29442568
Conv-18	BA.1	S:A67V,S:T95I,S:Y145D,S:L212I,S:G339D,S:S371L,S:S373P,S:S375F,S:K417N,S:N440K,S:G446S,S:S477N,S:T478K,S:E484A,S:Q493R,S:G496S,S:Q498R,S:N501Y,S:Y505H,S:T547K,S:D614G,S:H655Y,S:N679K,S:P681H,S:N764K,S:D796Y,S:N856K,S:Q954H,S:N969K,S:L981F	S:H69-,S:V70-,S:G142-,S:V143-,S:Y144-,S:N211-	ON897715
Conv-21	BA.1	S:A67V,S:T95I,S:Y145D,S:L212I,S:V320I,S:G339D,S:S371L,S:S373P,S:S375F,S:K417N,S:N440K,S:G446S,S:S477N,S:T478K,S:E484A,S:Q493R,S:G496S,S:Q498R,S:N501Y,S:Y505H,S:T547K,S:D614G,S:Q628K,S:H655Y,S:N679K,S:P681H,S:N764K,S:D796Y,S:N856K,S:Q954H,S:N969K,S:L981F	S:H69-,S:V70-,S:G142-,S:V143-,S:Y144-,S:N211-	ON897722
Conv-23	B.1	S:D614G		OM000280
Conv-24	B.1	S:D614G		OM000281
Conv-25	B.1	S:D614G		OM000284
Conv-26	B.1	S:D614G		OM000285
Conv-27	B.1	S:D614G		OM000282
Conv-28	B.1.2	S:D614G		OM000296
Conv-29	B.1	S:D614G		OM000286
Conv-30	B.1	S:D614G		OM000279
Conv-31	B.1	S:D614G		OM000288
Conv-32	B.1	S:D614G		OM000287
Conv-33	B.1	S:D614G		OM000283
Conv-34	B.1.2	S:D614G		OM000297
Conv-35	B.1.2	S:D614G		OM000299
Conv-36	B.1.2	S:D614G		OM000298
Conv-37	B.1.2	S:D614G		OM000294
Conv-38	B.1.1.207	S:D614G,S:P681H		ON897726
Conv-39	B.1.1.207	S:E484K,S:D614G,S:P681H		ON897727
Conv-40	B.1.1.207	S:E484K,S:D614G,S:P681H		ON897728
Conv-41	B.1.2	S:G257D,S:D614G		OM000295
Conv-42	P.1.10	S:L18F,S:T20N,S:P26S,S:D138Y,S:R190S,S:K417T,S:E484K,S:N501Y,S:D614G,S:H655Y,S:S704L,S:T1027I,S:A1078S,S:V1176F		ON897729
Conv-43	B.1.1.519	S:L5F,S:T478K,S:D614G,S:P681H,S:T732A		ON897730
Conv-44	B.1.526	S:L5F,S:T95I,S:D253G,S:E484K,S:D614G,S:A701V		ON897731
Conv-45	B.1.1.7	S:N501Y,S:A570D,S:D614G,S:P681H,S:T716I,S:Q836*,S:S982A,S:D1118H	S:H69-,S:V70-,S:Y144-,S:I870-,S:A871-,S:Q872-,S:Y873-,S:T874-,S:S875-,S:A876-,S:L877-,S:L878-,S:A879-,S:G880-,S:T881-,S:I882-,S:T883-,S:S884-,S:G885-,S:W886-,S:T887-,S:F888-,S:G889-,S:A890-,S:G891-,S:A892-,S:A893-,S:L894-,S:Q895-,S:I896-,S:P897-,S:F898-,S:A899-,S:M900-,S:Q901-,S:M902-,S:A903-,S:Y904-,S:R905-,S:F906-,S:N907-,S:G908-,S:I909-,S:G910-,S:V911-,S:T912-,S:Q913-,S:N914-,S:V915-,S:L916-,S:Y917-,S:E918-,S:N919-,S:Q920-,S:K921-,S:L922-	OM000289
Conv-46	B.1.1.7	S:N501Y,S:A570D,S:D614G,S:P681H,S:T716I,S:S982A,S:D1118H	S:H69-,S:V70-,S:Y144-	OM000292
Conv-47	B.1.1.7	S:N501Y,S:A570D,S:D614G,S:P681H,S:T716I,S:S982A,S:D1118H,S:K1191N	S:H69-,S:V70-,S:Y144-	OM000291
Conv-48	B.1.429	S:S13I,S:T95I,S:W152C,S:L452R,S:D614G		ON897732
Conv-49	B.1.429	S:S13I,S:W152C,S:L452R,S:D614G		ON897733
Conv-51	AY.74	S:T19R,S:G142D,S:R158G,S:A222V,S:L452R,S:T478K,S:D614G,S:P681R,S:D950N	S:E156-,S:F157-	OM000262
Conv-52	AY.62	S:T19R,S:G142D,S:R158G,S:A222V,S:L452R,S:T478K,S:D614G,S:P681R,S:G946V,S:D950N	S:E156-,S:F157-	OM000270
Conv-53	AY.47	S:T19R,S:G142D,S:R158G,S:A222V,S:V289I,S:L452R,S:T478K,S:D614G,S:P681R,S:D950N	S:E156-,S:F157-	OM000264
Conv-54	AY.25.1	S:T19R,S:G142D,S:R158G,S:L452R,S:T478K,S:D614G,S:P681R,S:D950N	S:E156-,S:F157-	OM000271
Conv-55	AY.14	S:T19R,S:G142D,S:R158G,S:L452R,S:T478K,S:D614G,S:P681R,S:D950N	S:E156-,S:F157-	OM000267
Conv-56	AY.14	S:T19R,S:G142D,S:R158G,S:L452R,S:T478K,S:D614G,S:P681R,S:D950N	S:E156-,S:F157-	OM000266
Conv-57	AY.3	S:T19R,S:G142D,S:R158G,S:L452R,S:T478K,S:D614G,S:P681R,S:D950N	S:E156-,S:F157-	OM000274
Conv-59	B.1.617.2	S:T19R,S:K77T,S:G142D,S:R158G,S:G181V,S:L452R,S:T478K,S:D614G,S:A653V,S:P681R,S:D950N	S:E156-,S:F157-	OM000265
Conv-60	B.1.617.2	S:T19R,S:K77T,S:G142D,S:R158G,S:G181V,S:L452R,S:T478K,S:D614G,S:A653V,S:P681R,S:D950N	S:E156-,S:F157-	OM311576
Conv-61	AY.25	S:T19R,S:S112L,S:G142D,S:R158G,S:L452R,S:T478K,S:D614G,S:P681R,S:D950N	S:E156-,S:F157-	OM000263
Conv-62	AY.25	S:T19R,S:S112L,S:G142D,S:R158G,S:L452R,S:T478K,S:D614G,S:P681R,S:D950N	S:E156-,S:F157-	OM000268
Conv-63	AY.44	S:T19R,S:T22I,S:G142D,S:R158G,S:L452R,S:T478K,S:D614G,S:P681R,S:D950N	S:E156-,S:F157-	OM000272
Conv-64	AY.100	S:T19R,S:T95I,S:G142D,S:R158G,S:L452R,S:T478K,S:D614G,S:P681R,S:D950N	S:E156-,S:F157-	OM000276
Conv-65	AY.119	S:T19R,S:T95I,S:G142D,S:R158G,S:L452R,S:T478K,S:D614G,S:P681R,S:D950N	S:E156-,S:F157-	OM000273
Conv-66	AY.100	S:T19R,S:T95I,S:G142D,S:R158G,S:L452R,S:T478K,S:D614G,S:P681R,S:D950N,S:G1124V	S:E156-,S:F157-	OM000269

Table S8. SARS-CoV-2 variant spikes of human primary infection serum samples commercially obtained

Specimen ID	Infecting Genotype	Spike mutations
738741	B.1	S:S13I;S:Q52R;S:A67V;S:L452R
743259	B.1	D614G
718055	B.1.2	D614G
718057	B.1.2	S24L;S:D614G
743136	B.1.234	D614G
743256	B.1.429	S:W152C;S:L452R;S:D614G
743257	B.1.429	S:L452R;S:D614G
743264	B.1.429	S:S13I;S:W152C;S:L452R;S:D614G
719166	B.1.577	D614G
743255	C.11	S:L452R;S:D614G
D000113656	B.1.1.7	S:N501Y;S:A570D;S:D614G;S:P681H;S:T716I;S:S982A;S:D1118H
D000113657	B.1.1.7	S:N501Y;S:A570D;S:D614G;S:P681H;S:T716I;S:S982A;S:D1118H
D000113667	B.1.1.7	S:V433F;S:A570D
D000113669	B.1.1.7	S:N501Y;S:A570D;S:D614G;S:P681H;S:T716I;S:S982A;S:D1118H
D000113675	B.1.1.7	S:V193L;S:W436*;S:V510L;S:A1020S;S:P1079S;S:D1118H
D000113694	B.1.1.7	S:N501Y;S:A570D;S:D614G;S:P681H;S:T716I;S:S982A;S:D1118H;S:P1263L
D000117099	C.37	S:G75V;S:T76I;S:R246N;S:L452Q;S:F490S;S:D614G;S:T859N
D000117104	C.37	S:G75V;S:T76I;S:R246N;S:L452Q;S:F490S;S:D614G;S:T859N
D000117136	C.37	S:G75V;S:T76I;S:R246N;S:D442Y;S:L452Q;S:F490S;S:Q580X;S:T581X;S:D614G;S:I714V
D000117154	C.37	S:G75V;S:T76I;S:R246N;S:L452Q;S:F490S;S:D614G;S:T859N
D00011366	B.1.1.7	S:N501Y;S:A570D;S:P681H;S:T716I;S:S982A;S:D1118H
D00012307	P.1	S:E484K;S:N501Y;S:D614G;S:H655Y;S:Q677R;S:T1027I;S:V1176F
D00012308	C.37	S:G75V;S:T76I;S:R246N; S:S247-;S:Y248-;S:L249-;S:T250-;S:P251-;S:G252-;S:D253-;S:L452Q;S:D614G;S:Q675H;S:I720V;S:T859N;S:P863L;S:Q1180
D00012308	C.37	S:G75V;S:T76I;S:R246N; S:S247-;S:Y248-;S:L249-;S:T250-;S:P251-;S:G252-;S:D253-;S:L452Q;S:F490S;S:D614G;S:I714V;S:T859N;S:G1219C
D00012308	C.37	S:W64-;S:H66-;S:A67-;S:I68-; S:T63X;S:F65X;S:H69-;S:V70-;S:S71-;S:G72-;S:T73-;S:N74-;S:G75-;S:T76-; S:D138Y;S:R246N;S:S247-;S:Y248-;S:L249-;S:T250-;S:P251-;S:G252-;S:D253- S:L452Q;S:F490S;S:D614G;S:T859N
D000123091	C.37	S:G75V;S:T76I; S:S247-;S:Y248-;S:L249-;S:T250-;S:P251-;S:G252-;S:D253-S:R246N;S:L452Q;S:D614G;S:T859N
D000123095	C.37	S:G75V;S:T76I; S:S247-;S:Y248-;S:L249-;S:T250-;S:P251-;S:G252-;S:D253-;S:R246N;S:L452Q;S:F490S;S:D614G;S:T859N
D000123099	C.37	;S:G75V;S:T76I; S:S247-;S:Y248-;S:L249-;S:T250-;S:P251-;S:G252-;S:D253-;S:R246N;S:L452Q;S:F490S;S:D614G;S:T859N
D000123108	P.1	S:D138Y;S:R190S;S:K417T;S:E484K;S:N501Y;S:D614G;S:H655Y;S:Q677R;S:T1027I;S:V1176F
D000123182	P.1	S:L18F;S:T20N;S:P26S;S:D138Y;S:R190S;S:K417T;S:E484K;S:N501Y;S:D614G;S:H655Y;S:T1027I;S:V1176F
D000123194	P.1	S:D138Y;S:K417T;S:E484K;S:N501Y;S:D614G;S:H655Y;S:T1027I;S:V1176F
